# Mesenchymal Stem/Stromal Cells in Progressive Fibrogenic Involvement and Anti-Fibrosis Therapeutic Properties

**DOI:** 10.3389/fcell.2022.902677

**Published:** 2022-06-01

**Authors:** Chenghai Li, Bin Wang

**Affiliations:** ^1^ Stem Cell Program of Clinical Research Center, People’s Hospital of Zhengzhou University and Henan Provincial People’s Hospital, Zhengzhou, China; ^2^ Henan Key Laboratory of Stem Cell Differentiation and Modification, Henan University, Zhengzhou, China; ^3^ Department of Neurosurgery, People’s Hospital of Zhengzhou University and Henan Provincial People’s Hospital, Zhengzhou, China

**Keywords:** fibrosis, instant blood-mediated inflammatory reaction, mesenchymal stem/stromal cell, fibrotic mesenchymal cells, stem cell differentiation

## Abstract

Fibrosis refers to the connective tissue deposition and stiffness usually as a result of injury. Fibrosis tissue-resident mesenchymal cells, including fibroblasts, myofibroblast, smooth muscle cells, and mesenchymal stem/stromal cells (MSCs), are major players in fibrogenic processes under certain contexts. Acknowledging differentiation potential of MSCs to the aforementioned other types of mesenchymal cell lineages is essential for better understanding of MSCs’ substantial contributions to progressive fibrogenesis. MSCs may represent a potential therapeutic option for fibrosis resolution owing to their unique pleiotropic functions and therapeutic properties. Currently, clinical trial efforts using MSCs and MSC-based products are underway but clinical data collected by the early phase trials are insufficient to offer better support for the MSC-based anti-fibrotic therapies. Given that MSCs are involved in the coagulation through releasing tissue factor, MSCs can retain procoagulant activity to be associated with fibrogenic disease development. Therefore, MSCs’ functional benefits in translational applications need to be carefully balanced with their potential risks.

## Graphical Abstract

Pathophysiological and therapeutic implications of MSCs.
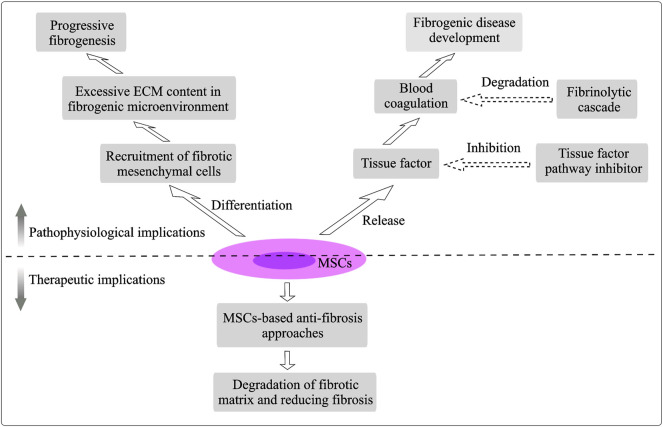

## Introduction

Fibrosis is characterized by the excessive deposition of extracellular matrix (ECM) components, including collagen, proteoglycan, fibronectin, etc. which appears in various tissues in the context of the both physiological tissue remodeling and pathological wound healing. Physiological fibrosis is protective in response to the injury. Pathological outcomes of progressive fibrosis due to a range of causes/triggers usually lead to fibrogenic scarring of tissues, subsequently impairing organ function, and eventually organ failure and death. Fibrotic diseases have been seen a major cause of morbidity and mortality in recent years especially in industrialized countries worldwide (Penke and Peters-Golden, 2019) and, however, no definitively effective treatment options have emerged to date. The progressive fibrogenesis orchestrates a complex biological process involving multiple cellular and molecular signaling pathways in response to fibrosis-associated pathological, physiological and environmental factors. A large variety of cellular populations, such as immune cells and inflammation cells, are being identified in fibrotic tissues to contribute to the fibrogenic process (Lenti et al., 2019; Penke and Peters-Golden, 2019; Weiskirchen et al., 2019). Mesenchyme-derived cell types, including fibroblasts, myofibroblasts, smooth muscle cells (SMCs), and mesenchymal stem/stromal cells (MSCs), are major intermediate/ultimate effectors of tissue fibrosis through secretion of pro-fibrotic mediators including excessive ECM content (Penke and Peters-Golden, 2019; Weiskirchen et al., 2019). MSCs play a distinct role in progressive fibrogenesis.

MSCs are multipotent progenitor cells and they can be induced *in vitro* to give rise to the differentiated cell types, including osteoblasts, chondrocytes and adipocytes (Kumar et al., 2017). MSCs are initially described in bone marrow but later found in wide variety of human tissues, including adipose tissue, skin, muscle, dental pulp, and the neonatal birth-associated tissue sources (Li et al., 2021), such as umbilical cord tissue, Wharton’s jelly, amniotic membrane and placenta. More than a half-century ago, MSCs were originally identified within rodent bone marrow by Friedenstein et al. (1966) and such cells were rapidly adhered to the glass surface and later formed the discrete “fibroblast” colonies (Friedenstein et al., 1966; Friedenstein et al., 1970). These “fibroblasts” from bone marrow are inducible to osteogenesis *in vitro* in the diffusion chambers. The name of “Mesenchymal stem cells” was coined by Caplan in the late 1980s (Caplan, 1991; Caplan, 2010) who discussed and emphasized several key facts that were evident: i) osteogenic and chondrogenic potential of embryonic mesenchymal cells; ii) lineage of mesenchymal cells; and iii) bioactive factors for self-cell repair of skeletal defects in bone. MSCs are actively in response to biological signals associated with inflammation, aging and tissue injury. As known, MSCs derived from different tissue sources present the morphological irregularities, varied phenotypic features, and functional heterogeneity (Costa et al., 2021; Li et al., 2021). The varied methodologies of cell preparation in different laboratories elicit the question of cell equivalence, especially in the context of cell therapy. To address these issues, the Mesenchymal and Tissue Stem Cell Committee of the International Society for Cellular Therapy (ISCT) proposes minimal criteria for universally defining in vitro-expanded MSCs (Dominici et al., 2006): i) MSCs’ plastic adherence; ii) expression of CD105, CD73, and CD90 but not CD45, CD34, CD14/CD11b, CD79α/CD19, and HLA-DR; and iii) trilineage differentiation potential (osteogenesis, chondrogenesis and adipogenesis). Later, Caplan urged to change the name of MSCs to Medicinal Signaling Cells to more accurately reflect the MSCs’ homing in sites of injury or disease and their secreting bioactive mediators (Caplan, 2010; Caplan, 2017). Given that MSCs do not seem to meet generally accepted criteria for stem cell activity, in 2005, the ISCT suggested MSCs to be termed multi-potential mesenchymal stromal cells (Horwitz et al., 2005). To date, the issue of term “stem cells” in MSCs nomenclature still remains contentious. As of 27 April 2022, there were 80286 references in PubMed to “mesenchymal stem cell” versus 82511 to “mesenchymal stromal cell”. In 2019, the ISCT MSCs Committee suggested the functional definition of mesenchymal stem versus stromal cells to further consolidate and clarify the nomenclature of MSCs (Viswanathan et al., 2019), which may more accurately reflect the therapeutic benefits of MSCs or MSC-based products.

In this review, we will firstly discuss regarding MSCs’ contribution to fibrogenic pathophysiological events under profibrogenic conditions and further analyze the underlying mechanisms of MSCs as a potential initiator of coagulation associated with the fibrogenic disease development. We will then summarize preclinical and clinical studies using of MSCs or MSC-derived products as an anti-fibrotic therapeutic option owing to their unique therapeutic properties. Finally, in discussion section, we will analyze potential therapeutic properties of MSCs and pose great challenges for their translational anti-fibrotic applications.

## Mesenchymal Stem/Stromal Cells in Contribution to Progressive Fibrogenesis

Primary lung-resident MSCs can be isolated from patients with idiopathic pulmonary fibrosis (IPF), a progressive lung disease of unknown etiology, and the increased number of MSCs accompanied by the augmented ECM deposition has been observed in the lung interstitium (Hostettler et al., 2017; Boesch et al., 2020). Lung-resident MSCs have been also shown to undergo phenotype conversion, for example, to myofibroblasts *in vivo*, and to increase ECM products contributing to pulmonary fibrogenesis (Chen et al., 2018; Cao et al., 2020). Acknowledging the cellular and molecular mechanism of MSCs’ action in fibrosis, it is essential to note that MSCs can contribute partially to the recruited fibrogenesis-associated mesenchymal cell populations, including fibroblasts, myofibroblasts and SMCs, through differentiation of MSCs under certain contexts. Other cells such as immune cells and inflammation cells that also contribute to progressive fibrogenesis will not be considered in this section. We focus on several points regarding potential contribution of MSCs in progressive fibrogenesis and this could be conducive to the mechanistic understanding of pathophysiological and anti-fibrotic implications of MSCs under different fibrogenic conditions.

### Fibroblasts in Tissue Fibrogenesis and Mesenchymal Stem/Stromal Cells to Fibroblasts Differentiation

MSCs are well known to be originated from perivascular cells and reside in perivascular niches of the multiple organs/tissues (Di Carlo and Peduto, 2018; Wang S et al., 2020; Steens et al., 2021). Perivascular MSC-like cells seem to have potential contribution to different types of tissue fibrosis under certain contexts, such as myocardial fibrosis, liver fibrosis, and kidney fibrosis (Kramann et al., 2015). Progressive tissue fibrogenesis involves the cross-talk of a multitude of cell types triggering and sustaining fibrosis. Fibroblasts, the principal cellular type of the connective tissues, have various roles including wound healing, immunoregulation, angiogenesis, aging, and neoplasia (LeBleu and Neilson, 2020; Buechler et al., 2021; Zou et al., 2021). The functional heterogeneity of fibroblasts is likely resident tissue-specific. Fibroblasts may be affected by fibrogenic conditions, inflammation, etc. and exhibit the capacity to express high levels of ECM content (Buechler et al., 2021). Importantly, fibroblasts have the potential of differentiation into SMCs by induction of DKK3, a member of the Dickkopt family of Wnt inhibitors, *via* activation of transforming growth factor (TGF)-β signaling (Karamariti et al., 2013; Karamariti et al., 2018), thus suggesting that fibroblasts are also involved in SMC-associated fibrosis (see below). MSCs are fibroblast-looking cells but not fibroblasts. Soundararajan and Kannan discussed regarding the similarities and differences between fibroblasts and MSCs in their systematic review and proposed that the both cells are the same (Soundararajan and Kannan, 2018). MSCs and fibroblasts share common features of morphology, cellular phenotype, growth and differentiation potential, *in vitro* immunoregulation, and gene expression profiles (Hematti, 2012; Spitzer et al., 2012). However, cellular markers that are uniquely expressed in fibroblasts are lacking to identify this specific population. The exact relationship between MSCs and fibroblasts remain undetermined.

One previous study conducted by Khatun et al. (2017) demonstrated that human bone marrow-derived MSCs (BM-hMSCs) and endometrial MSCs had the high proliferation and migration potential in response to inflammation compared with endometrial fibroblasts. *In vitro* data obtained from this study by Khatun and colleagues (Khatun et al., 2017) showed the distinct differences between BM-hMSCs and endometrial fibroblasts in their cytokine secretion profiles. Another *in vitro* study revealed that the endometrial MSCs can differentiate into endometrial stromal fibroblast lineage but showing kind of differentially expressed genes versus the fibroblast cultures (Barragan et al., 2016). Endometrial MSCs were further substantiated, as progenitors of endometrial fibroblasts, to differentiate into these endometrial stromal fibroblasts (Spitzer et al., 2012; Barragan et al., 2016). Previous *in vitro* studies also showed that fibroblastic differentiation of human adipose-derived MSCs (AD-hMSCs) was enhanced by bone morphogenetic protein-4 treatment under hypoxic culture conditions (Lui et al., 2021) and that dermal fibroblast differentiation of bone marrow MSCs was inhibited by suppressing ERK/β-catenin signaling (Cheng et al., 2021). Still, TGF-β could induce fibroblast differentiation of lung resident MSCs *in vitro* (Chen et al., 2016). Importantly, previous *in vivo* studies revealed that exogenous MSCs were capable of differentiation into lung fibroblasts and myofibroblasts through Wnt/β-catenin signaling in the fibrogenic environment of the injured lung after transplantation and this promoted pulmonary fibrogenesis (Sun et al., 2014a; Sun et al., 2014b).

### Myofibroblasts in Tissue Fibrosis and Myofibroblasts Differentiation of Fibroblasts and Mesenchymal Stem/Stromal Cells

Fibroblasts are not a terminally differentiated cell type and they are more likely to be of the cellular origin of myofibroblasts. Cardiac fibrosis is mediated by the activated resident fibroblasts that differentiate into highly specialized cardiac myofibroblasts in response to acute injury (Gibb et al., 2020). Profibrogenic mediators such as TGF-β and angiotensin II are involved in the transition process. One previous study showed that fibroblasts became activated and then were induced to differentiate into myofibroblasts (Fu et al., 2018). These myofibroblasts secreted abundant ECM proteins and expressed contractile genes such as α-smooth muscle actin (α-SMA) in the acute wound healing response after mouse myocardial infarction injury (Fu et al., 2018). Hepatic myofibroblasts can originate from portal fibroblasts that are mainly involved in conditions of biliary fibrosis (Parola and Pinzani, 2019). Previously, LeBleu et al. (2013) reported that myofibroblasts were functional contributor of type I collagen production in kidney fibrosis and MSCs could give rise to myofibroblasts *via* TGF-β1 dependent differentiation. Similarly, MSCs were treated *in vitro* with TGF-β1 and subsequently changed their gene expression indicative of myofibroblast profiles (Marriott et al., 2014). Gli1 is a marker of perivascular MSC-like cells and perivascular Gli1^+^ cells have been seen to proliferate and trans-differentiate into α-SMA positive myofibroblasts in the kidney and heart fibrosis by following organ injury (Kramann et al., 2015). As mentioned above, exogenous MSCs after transplantation under lung tissue fibrotic conditions may differentiate into myofibroblasts in response to lung injury and contribute to fibrogenesis (Sun et al., 2014a; Sun et al., 2014b). Another *in vivo* study demonstrated that lung resident MSCs could also differentiate into myofibroblasts in the development of pulmonary fibrosis. Cao et al. (2018) conducted this study to consider the ATP-binding cassette transporter subtype G 2 (ABCG2) as a marker for lung resident MSCs and they observed the transition of these MSCs from an ABCG2-expressing cellular phenotype to a myofibroblast phenotype in a bleomycin-induced mouse fibrosis model.

### Smooth Muscle Cells in Tissue Fibrosis and Mesenchymal Stem/Stromal Cells to Smooth Muscle Cells Differentiation

SMCs may proliferate and release excessive ECM content that significantly contributes progressive fibrogenesis (Mahavadi et al., 2011; Li et al., 2013; Moulton et al., 2018). Li and colleagues conducted one previous study that presented a mechanism by which leads to increased TGF-β1 activation of SMCs in the intestinal structures, which develops in a certain percentage of patients affected with Crohn’s disease (Li et al., 2013). The increase levels of activated TGF-β1 caused the excessive deposition of ECM component (collagen I) and development of fibrosis in Crohn’s disease. Similarly, in an experimental study, Mahavadi et al. (2011) showed that insulin-like growth factor-I (IGF-I) caused SMC hyperplasia and collagen production, resulting in fibrosis in colitis. Still, an early study indicated that SMCs-specific phosphatase and tensin homolog (PTEN) deficiency in mice led to spontaneous vascular fibrosis (Nemenoff et al., 2008). Furthermore, the loss of PTEN in SMCs promoted vascular fibrosis in human coronary arteries from patients implanted with continuous-flow left ventricular assist devices (Moulton et al., 2018). It was previously noted that mature SMCs were able to be reprogrammed into a subpopulation of resident vascular progenitor cells, AdvSca1-SM cells, in the adventitia by induction of transcription factor Klf4 (Majesky et al., 2017; Lu et al., 2020). Surprisingly, SMC-derived AdvSca1-SM progenitor cells downregulated the level of Klf4 and subsequently differentiated multiple cell types including profibrogenic myofibroblasts in response to vascular injury. AdvSca1-SM-derived myofibroblasts exhibited a robust fibrogenic response (Lu et al., 2020). Most importantly, one previous study showed that adventitial MSC-like cells are progenitors of vascular SMCs (Kramann et al., 2016). Thus, the finding of a Klf4-dependent SMCs reprogramming process will broaden our understanding of MSCs’ contribution to pathophysiological adventitial remodeling and fibrosis.

Previously published data showed that AD-hMSCs underwent differentiation into SMCs with or without TGF-β1 (Wang et al., 2010; Zhang R et al., 2012; Park et al., 2013). Park and colleagues conducted an experimental study to show TGF-β1-induced differentiation of AD-hMSCs to SMCs (Park et al., 2013). Expression of SMC-specific marker proteins (e.g., α-SMA, calponin, smoothelin-B, myocardin, and h-caldesmon) in AD-hMSCs was observed after exposure of AD-hMSCs to TGF-β1 (Park et al., 2013). AD-hMSCs were induced with TGF-β1 together with bone morphogenetic protein-4 to express the SMC-related early and mid markers (α-SMA, SM22α, calponin) and the late marker (SM myosin heavy chain) (Wang et al., 2010). Ding et al. (2020) demonstrated that, for optimal setup for MSC-derived SMC maturation, combining biomimetic matrix stiffness and tethered TGF-β1 on the poly (ethylene glycol) hydrogels enhanced the potency of vascular SMCs commitment from human MSCs *in vitro* and *in vivo*. The 3-D culture of MSCs on specific biomaterials improved maturity of stem cell and, therefore, the study by Ding et al. (2020) further support the mechanical stimulation playing a potential role in MSCs’ differentiation into mature SMCs. Recently, one study was published that MSCs were cultured on the ultrathin nanostructured arrays patterned by self-assembly of grapheme oxide sheets and these aligned MSCs on patterned substrate surface were induced to differentiate into SMCs (Park et al., 2022). A previous study was conducted by Zhang R. et al. (2012) to evaluate the effect of a smooth muscle environment in the SMC differentiation of AD-hMSCs *in vitro* and *in vivo*. *In-vitro* experimental results indicated that coculturing of AD-hMSCs with rat bladder SMCs promoted SMC differentiation of AD-hMSCs. Likewise, *in vivo* evaluation in this study by Zhang R et al. (2012) showed a time-dependent SMC differentiation of AD-hMSCs injected into the smooth musculature of the urinary bladder. The study by Zhang R et al. (2012) is to emphasize the importance of the local environment in SMC differentiation of AD-hMSCs and it also suggests a novel therapeutic approach to repair smooth muscle.

**FIGURE 1:**
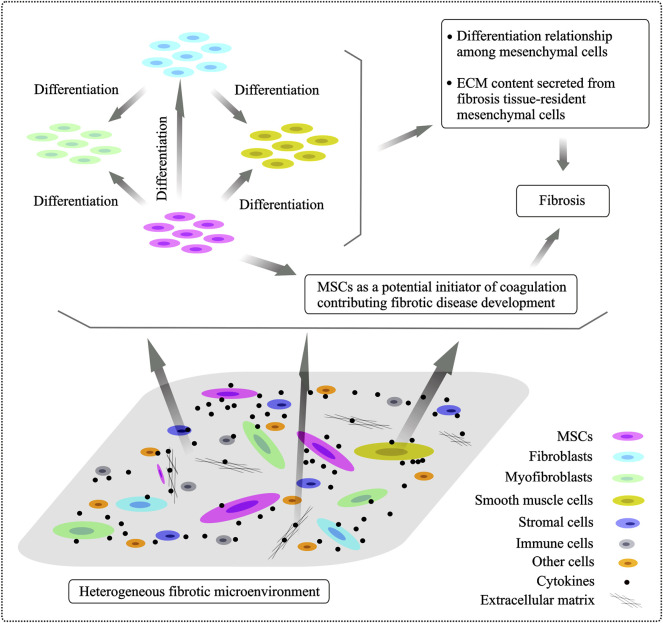
Heterogeneity of fibrogenic cells involved in the fibrogenic processes and differentiation relationship of mesenchymal cells. The fibrotic niche of microenvironment is very heterologous and it is composed of various cellular subpopulations and stromal entities. Resident fibrotic tissue-associated mesenchyme-derived cell types, including fibroblasts, myofibroblasts, smooth muscle cells and MSCs, are major players in tissue fibrosis development. Transformation relationship among mesenchymal cells localized in the fibrotic tissues is supposed and, specially, MSCs have been shown to undergo differentiation into other mesenchymal cells under different conditions, playing a distinct role in progressive fibrogenesis.

As aforementioned, MSCs undergo differentiation into the other lineage-restricted cell types, which proposes an important mechanism of MSCs’ action in tissue fibrosis. MSCs’ crosstalk with other fibrotic tissue-resident mesenchymal cells, including fibroblasts, myofibroblasts and SMCs, in a heterogeneous fibrogenic microenvironment is summary in Figure 1.

### Plasticity of Mesenchymal Stem/Stromal Cells in Fibrogenic Responses in the Heterogeneous Contexts

MSCs are at the crossroad of contribution to pathological fibrosis on the basis of certain contexts. An early *in vivo* study demonstrated that PDGFRα^+^ cells (i.e., MSCs) are derived mainly from pre-existing PDGFRα^+^ cells in the fibrotic environment and the expression of TGF-β and its isoforms was significantly upregulated (Uezumi et al., 2011). Importantly, the fibrosis-related molecules (collagen type I and α-SMA) under pathogenic conditions were upregulated in these PDGFRα^+^ cells in response to TGF-β (Uezumi et al., 2011). Santini et al. (2020) suggested a context- and spatio-temporal dependent “yin-yang” property of tissue-resident PDGFRα^+^ cells contributing to fibrosis versus regeneration. These progenitor PDGFRα^+^ cells played a double-edge role, as showed on the one hand stabilize newly formed blood vessels for maintenance of physiological niche homeostasis and on the other hand promote fibrosis in an unfavorable environment (Santini et al., 2020). The normal and pathological healing can be expanded to imply MSCs’ biology can be impacted by the pro-fibrogenic microenvironment.

## Mesenchymal Stem/Stromal Cells as a Potential Initiator of Coagulation Contributing Partially to Fibrogenic Disease Development

After learning more about contribution of MSCs to progressive fibrogenesis, there is still much more to be learnt about stem cell science and medicine. MSCs as a potential initiator of coagulation appear to be the incomplete understanding in MSC-based therapies. As such, the detrimental functional consequences in MSCs’ medical practices may hinder their therapeutic safety and effectiveness and, therefore, MSCs should be used with great caution in the clinical settings.

### Procoagulant Activity of MSCs

Mounting evidence is emerging regarding the procoagulant effect of MSCs through the release of tissue factor (TF) (Christy et al., 2017; Moll et al., 2019), which could increase the risk of clot formation. TF can be induced in MSCs *in vivo* by inflammatory mediators as well as *in vitro* by the increased passages of cells during culture (Witkowski et al., 2016; Caplan et al., 2019; Moll et al., 2019) and different MSCs’ tissue sources display highly variable expression of TF (Oeller et al., 2018; Moll et al., 2019). For example, one previous study showed the TF expression by the majority of AD-hMSCs and human umbilical cord-derived MSCs (UC-hMSCs) compared to <7% of BM-hMSCs expressing TF (Oeller et al., 2018). TF acts as a potential activator of the extrinsic blood coagulation cascade by initiating thrombin formation from the zymogen prothrombin (Witkowski et al., 2016; Moll et al., 2019). TF is the factor VII receptor and the complex TF:VIIa activates the coagulation factor X and IX to lead to thrombin generation and subsequently fibrin and thrombus formation (Witkowski et al., 2016; George et al., 2018). Therefore, the TF/FVIIa complex may be as a potential antithrombotic target for the future preclinical and clinical investigations. Culture-expanded MSCs may elicit an innate immune attack, such as complement activation and coagulation, termed instant blood-mediated inflammatory reaction (IBMIR) after blood exposure *in vitro* and *in vivo* (Moll et al., 2012; Moll et al., 2019).

Previously, Moll et al. (2012) conducted an experimental study that blood exposure of MSCs lead to initiation of the coagulation cascade *in vitro*, as showed the increased formation of thrombin and clotting factors such as activated FVIIa, FXIa, and FXIIa. Clinical data presented by Moll et al. (2012) from 44 MSCs recipients for treatment of life-threatening complications to hematopoietic stem cell transplantation suggested that intravenous infusion of MSCs elicits a weak triggering of IBMIR. Importantly, the IBMIR was to be likely dependent on the variability of MSCs’ properties such as the high cell doses, the individual MSCs donors, and the high cellular passage numbers. One previous study (Oeller et al., 2018) demonstrated that culture-expanded BM-hMSCs with significantly lower TF expression showed the lack of pro-coagulant activity *in vitro* compared to highly TF-expressing AD-hMSCs and UC-hMSCs through testing of the thromboelastometry parameters. Injection of TF-deficient BM-hMSCs at a dose of 6 × 10^6^ cells/kg body weight per rat also showed the lack of intravascular clot formation while intravascular thromboembolism was observed by immunohistochemical staining in lung, liver and spleen after injection of TF-expressing UC-hMSCs. Similarly, Liao et al. (2017) reported intravenous injection of TF-expressing mouse bone marrow MSCs into mice could induce disseminated micro-thrombi in the heart, liver, kidney and spleen. Anticoagulation treatment by heparin by 400 U/kg could prevent MSCs-induced coagulation in a colitis mouse model. *In vitro* coagulation assay showed the increased procoagulant activity of bone marrow MSCs derived from mouse, human, and goat during the expansion, respectively (Liao et al., 2017). Notably, the above two *in vivo* studies also suggested that a large amount of exogenous cells could significantly increase clot formation. IBMIR is a detrimental instant innate immune attack, thus suggesting that, from a safety perspective, optimizing MSCs’ therapeutic regimens, for example, testing TF expression, selecting TF-deficient MSCs, a low dose and a low passage number, needs to stress to avoid triggering the IBMIR.

### Coagulation and Fibrogenic Disease Development

Given that MSCs are involved in the coagulation through releasing TF, a central unanswered question concerns whether MSCs retain procoagulant activity to be associated with fibrogenic disease development. Mounting evidence indicates a frequent incidence of the prothrombotic state in patients with IPF (Navaratnam et al., 2014; Mattoo and Pillai, 2021). Navaratnam et al. (2014) conducted a population-based case-control study to propose a strong association between a prothrombotic state and fibrosis and, in this study, they enrolled 211 incident cases of IPF and 256 age- and sex-matched population-based controls. Compared to the controls, patients with IPF were more than four times likely to have a prothrombotic state that was associated with disease severity and had a threefold increased risk of death among cases with IPF. This study by Navaratnam et al. (2014) suggests a biomarker of coagulation as a potential therapeutic target for IPF. Another clinical observational study reported that the microparticle-bound TF activity, in contrast, was significantly higher in patients with pulmonary fibrosis and was inversely correlated with lung function in those patients (Novelli et al., 2014). While an association between a prothrombotic state and IPF was supposed, the manipulation of the coagulation cascade may be a potential therapeutic strategy for patients with IPF. However, clinical data from anticoagulation in IPF presented partly conflicting results in other clinical investigations (Abe et al., 2015; Kreuter et al., 2016). In addition, a prothrombotic state frequently appears in different tissue/organ types of fibrosis not only in pulmonary fibrosis but also in hepatic fibrosis (Joshi et al., 2015; Pant et al., 2018). The impact of coagulation in liver fibrogenesis and fibrosis development is also transparent and, however, the divergent results are presented in anticoagulation therapy for hepatic fibrosis (Bitto et al., 2018). Therefore, anticoagulation therapy in fibrosis needs validation from large studies.

### Fibrinolytic Activity of Mesenchymal Stem/Stromal Cells

**FIGURE 2:**
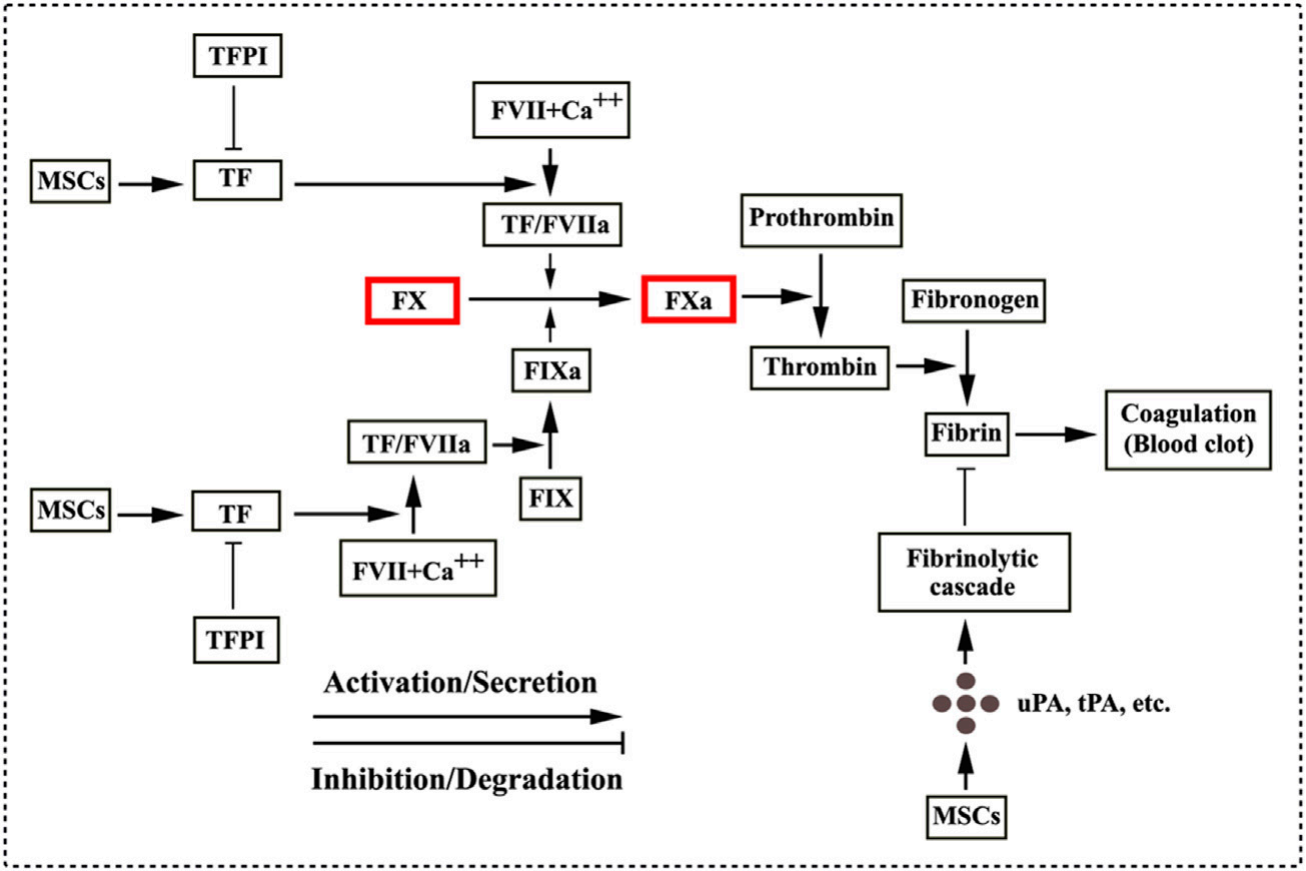
MSCs involved in coagulation and fibrinolytic cascade. MSCs/MSC-derived extracellular vesicles release tissue factor (TF) that initiates the coagulation cascade through forming a complex of TF/FVIIa. The TF/FVIIa complex converts FX to its active form FXa and FIX to FIXa (Witkowski et al., 2016; George et al., 2018). Subsequently, a coagulation cascade proceeds as a series of proteolytic reactions. FXa catalyses the conversion of the inactive plasma prothrombin to its active form thrombin, which in turn converts plasma fibrinogen into fibrin. Treads of fibrin trap red blood cells to form blood clots. TF activity is regulated by TF pathway inhibitor (TFPI) (Morrissey, 2001; Adams, 2012; Zelaya et al., 2018), seeming to be potentially effective in decreasing thrombosis. MSCs are also involved in the fibrinolytic cascade by the secretory production from MSCs such as the urokinase plasminogen activator (uPA), the tissue plasminogen activator (tPA) and the plasminogen activator inhibitor in context of pathological conditions (Neuss et al., 2010; Acosta et al., 2013).

TF can function as a cellular “signaling receptor” (Morrissey, 2001; Zelaya et al., 2018) and the activity of TF is regulated by TF pathway inhibitor (TFPI) in blood (Adams, 2012; Morrissey, 2001; Zelaya et al., 2018). Additionally, an early *in vitro* study demonstrated that the cultured fibrin-embedded human MSCs expressed the plasminogen activators, such as uPA and tPA, and the plasminogen activator inhibitor (PAI) (Neuss et al., 2010), thus suggesting that MSCs are possibly involved a fibrinolytic cascade in a context dependent manner. A previous clinical trial was conducted in a cohort of 30 diabetic patients (type 1 and 2) with critical limb ischemia and peripheral microthrombosis was observed in two type 2 diabetic patients after autologous AD-hMSCs administration (Acosta et al., 2013). Furthermore, AD-hMSCs derived from the type 2 diabetic patients exhibited the decreased serum-independent fibrinolytic activity (Acosta et al., 2013). As aforementioned, MSCs play a dual role in the triggering of IBMIR that is involved by the TF production secreted from MSCs and in the fibrinolytic cascade associated with the expression of the active fibrinolytic enzymes from MSCs under certain contexts. A potential trigger of the TF pathway of coagulation and a fibrinolytic cascade are summarized in Figure 2.

## Preclinical and Clinical Applications of Mesenchymal Stem/Stromal Cells in Fibrosis

Given their unique biological properties of the paracrine activity, immunomodulatory functions, multipotent differentiation potential and the lacking of immunogenicity, MSCs have drawn attention for stem cell-based therapy in the translational biomedicine field. The therapeutic potential of MSCs in fibrosis has been observed in preclinical models and clinical trials.

### Anti-Fibrotic Approaches of Using Mesenchymal Stem/Stromal Cells or Mesenchymal Stem/Stromal Cells Derived Products in Preclinical Studies

MSCs have been demonstrated to improve tissue functional recovery and ameliorate adverse fibrosis. Anti-fibrotic effects of MSCs-based therapies in preclinical studies are widely described in a variety of types of fibrosis models by Usunier et al. in their systematic review, such as cardiac fibrosis, liver cirrhosis, renal interstitial fibrosis, pulmonary fibrosis, fibrosis in the pancreas, and cutaneous fibrosis (Usunier et al., 2014). Nevertheless, MSCs are short-lived after systemic intravenous administration and the role of MSCs in engraftment, proliferation and differentiation *in vivo* is a subject of active debate (Parekkadan and Milwid, 2010; von Bahr et al., 2012). Several different therapeutic approaches in preclinical models have been described in this section to develop MSC-based anti-fibrosis applications.

#### Beneficial Effects of the “Natural” Mesenchymal Stem/Stromal Cells

Hepatic fibrogenesis is caused by the excessive deposition of collagen content and the collagen-producing myofibroblasts play a significant role in liver fibrotic processes (van der Helm et al., 2019). Liver fibrosis may develop into cirrhosis and liver failure in many clinical cases (Lim et al., 2017). Therapeutic benefits of the natural MSCs, i.e., unmodified MSCs, for liver fibrosis were observed in previous *in vivo* studies (Luo et al., 2019; van der Helm et al., 2019). For example, a previous study reported that liver fibrosis was reversed by local administration of MSCs in a cirrhotic mouse model (van der Helm et al., 2019). The study conducted by van der Helm et al. (2019) showed that, compared to the control without MSCs’ treatment, a significant reduction of collagen content and liver function improvement were observed in the locally treated lobes of liver in the mouse model. Underlying capacity of MSCs for further enhancing tissue remodeling has been proposed to differentiate into hepatocytes, to inhibit collagen-producing myofibroblasts, and to secrete a variety of cytokines and growth factors (e.g., hepatocyte growth factor, HGF). Anti-fibrotic potential of the unmodified MSCs has been reported in other animal models of different tissue types of fibrosis, such as diabetic lung fibrosis (Chen Y. et al., 2020), skin fibrosis (Maria et al., 2018), ovarian fibrosis (Cui et al., 2020), intestinal fibrosis (Choi Y. et al., 2019), and injury-induced corneal fibrosis (Shukla et al., 2019).

#### Conditioned Medium Derived From Mesenchymal Stem/Stromal Cells

Given the MSCs’ paracrine properties, CM obtained from MSCs (MSCs CM) during culture containing a complex of MSC-secreted products is being explored in the current translational medicine. One previous *in vitro* study showed that MSCs CM had anti-fibrotic potential effects through downregulation of collagen I, collagen III and α-SMA in fibroblasts derived from hypertrophic scar (Chai et al., 2019). Preclinical data from silica-induced pulmonary fibrogenic animal model presented that collagen I, collagen III, fibronectin were decreased in the MSCs CM-treated rats (Li et al., 2018). Hu et al. conducted one previous preclinical study to compare different therapeutic effects on wound healing and hypertrophic scar formation in an animal model treated with MSCs CM, bone marrow concentrate (BMC) CM, or BMC-induced MSCs CM (Hu et al., 2019). Experimental results indicated that both MSCs CM and BMC-induced MSCs CM had therapeutic effects on preventing hypertrophic scar formation, as showed that the treated fibrogenic scars resulted in much lower scar elevation index, a method of measurement of the collagen fiber arrangement by immunohistochemical staining, compared to control samples. Interestingly, BMC-induced MSCs CM had greater anti-fibrotic effects than MSCs CM (Hu et al., 2019). However, the specified therapeutic factors in MSCs CM are unclear and the exact mechanism has yet to be fully elucidated.

Specifically, HGF-expressing MSCs were shown to attenuate bleomycin induced pulmonary fibrosis in an early preclinical study conducted by Gazdhar et al. (2013). Based on this study, the same team conducted a subsequent clinical study performed on different lung tissues from patients with fibrotic lung diseases, from emphysema, and from normal lungs (Hostettler et al., 2017). Clinical data presented by Hostettler et al. (2017) suggested that MSCs CM inhibited fibroblast proliferation and facilitated HGF-mediated lung epithelium wound healing. Importantly, they provided further evidence that the increased numbers of MSCs were obtained in adult fibrotic lungs compared to the non-fibrotic lungs. Treatments of MSCs or MSCs CM in a rat model of pulmonary fibrosis showed the increased expression of epithelial markers, E-cadherin and cytokeratin 19, and the declined expression of fibrosis mesenchymal markers, vimentin and α-SMA (Zhang et al., 2018), suggesting that MSCs or MSCs CM may help reduce fibrosis *via* inhibition of the epithelial to mesenchymal transition (EMT) program.

#### Pre-Licensing Mesenchymal Stem/Stromal Cells

Pre-licensing MSCs, for example, hypoxia-priming and pretreatment with cytokines, etc. can improve their anti-fibrotic therapeutic effect. One previous preclinical study indicated that the antioxidant preconditioning MSCs improved their anti-fibrotic therapeutic outcomes in an animal liver fibrosis model. This study was conducted by Liao et al. (2020) using the autologous adipose tissue-derived MSCs with and without antioxidant pretreatment. Study data showed the pretreatment with antioxidant in MSCs improved therapeutic effects of MSCs on liver function recovery in liver fibrosis mice through reducing oxidative stress-induced injury in MSCs and, responsibly, the increased intrahepatic engraftment of these MSCs (Liao et al., 2020). Similarly, two preclinical studies reported that hypoxia-preconditioned MSCs can prevent renal fibrosis in rats with ischemia-reperfusion injury (Ishiuchi et al., 2020) and pulmonary fibrosis in bleomycin-induced pulmonary fibrotic mice (Lan et al., 2015). In addition, MSCs pretreated with oncostatin M, a cytokine with both pro- and anti-inflammatory actions, were shown to enhance the therapeutic effectiveness of pulmonary fibrosis by the mechanisms involving the upregulation of the secreted anti-fibrotic factor, HGF, and the attenuation of TGF-β1-induced ECM production in lung fibroblasts (Lan et al., 2017). Still, administration of autologous MSCs pretreated with interferon (IFN)-γ, compared to MSCs without IFN-γ pretreatment, was observed to improve interstitial fibrosis in the unilateral ureter obstruction (UUO) rat models, as showed the decreased fibrotic proteins, α-SMA, collagen I and III (Kanai et al., 2021). Furthermore, the increased secretion of prostaglandin E2 were also observed in CM from IFN-γ preconditioned MSCs (Kanai et al., 2021).

#### Mesenchymal Stem/Stromal Cells-Derived Extracellular Vesicles

Due to the physical properties of MSCs, exogenous MSCs’ engraftment *in vivo* after infusion seems to be rare at the site of injury, thus hampering MSCs’ therapeutic efficacy. To avoid cell-related problems (e.g., spontaneous transformation of MSCs), exploring novel applications of MSC-derived EVs has been shown an effective treatment option for tissue fibrosis in recent years. EVs comprise exosomes, micro-vesicles and apoptotic bodies and they can carry a variety of substance including mRNAs, microRNAs, enzymes and other bioactive molecules (Abraham and Krasnodembskaya, 2020; Kanai et al., 2021). MSCs-derived EVs can be administered after isolation, characterization and purification from the CM of cultured MSCs.

Glial-derived neurotrophic factor (GDNF), a growth factor for human mesangial cells, was shown to promote proliferation and differentiation as well as the anti-inflammatory properties of MSCs in one previous study (Wang et al., 2019). GDNF-overexpressing MSCs significantly downregulated the expression of IL-6, cyclooxygenase-2, TGF-β1 and α-SMA in the renal tissue in a UUO animal model and upregulated the levels of IL-4 and IL-10 when GDNF-MSCs were cocultured with macrophages (Wang et al., 2019). Another previous *in vivo* study indicated that exosomes derived from GDNF-modified human MSCs (GDNF-MSCs-Exos) ameliorated peritubular capillary loss in tubulointerstitial fibrosis in a UUO mouse model (Chen L. et al., 2020). Preclinical data obtained from the above two studies suggest that GDNF-MSCs may exert their therapeutic effects through potential immunomodulatory properties for the repair of endogenous tissue damage caused by chronic inflammation, albeit the precise mechanisms of GDNF action in fibrosis improvement are not fully understood. Ji et al. (2020) carried out an experimental study that MSCs-Exos delivering casein kinase 1δ (CK1δ) and E3 ubiquitin ligase β-TRCP to degrade Yes-associated protein (YAP) ameliorated renal fibrosis in the rat UUO models. YAP plays an important role in fibrosis *via* the deposition of collagen content. Reversely, they conducted a further experimental study that showed silencing CK1δ and β-TRCP decreased the effects of the MSCs-Exos on renal fibrosis (Ji et al., 2020). These preclinical data suggest a new mechanism by which MSCs-Exos exert their therapeutic effects on renal fibrosis by the CK1δ/β-TRCP signaling that inhibits YAP activity, which provides a theoretical foundation for further exploration of MSCs-Exos-based anti-fibrotic therapies. The therapeutic effects of MSCs-EVs by a variety of mechanisms are being observed in other different types of tissue fibrosis in animals such as liver fibrosis (Qu et al., 2017), pulmonary fibrosis (Dinh et al., 2020) and corneal fibrosis (Shojaati et al., 2019). Xuan et al. (2020) conducted a previous *in vivo* study that EVs derived from Notch activated cardiac MSCs showed, compared to the control, to improve cardiac function, to increase neovasculogenesis, and to decrease fibrosis in a mouse myocardial infarction model. Significantly, proteomics profile was identified using mass spectrometry in EVs from these MSCs such as fibrillin-1/2, fibulin-2, lysyl oxidase homolog-2, biglycan, laminin subunit beta-1, etc. which are related to various functional features of MSCs.

#### Genetically Modified Mesenchymal Stem/Stromal Cells

Given the paracrine feature of MSCs, utilizing MSCs modified as potential carriers has been developing to deliver therapeutic genes/molecules, such as GDNF (mentioned above), HGF, vascular endothelial growth factor (VEGF), and IL-10. These bioactive modulators are implicated in their roles in tissue remodeling. The application of MSCs transfected with HGF was observed to significantly suppress dimethylnitrosamine-induced liver fibrosis and to improve liver function in rats (Moon et al., 2019). This study by Moon et al. (2019) demonstrated that HGF-transfected MSCs significantly decreased collagen fiber-occupied regions compared to the natural MSCs. Similarly, MSC-based VEGF gene therapy in the rat myocardial infarction model showed that, compared with the control, the VEGF-MSC treatment significantly attenuated left ventricular fibrosis in ischemic myocardium (Moon et al., 2014). Additionally, MSCs genetically modified to overexpress thioredoxin-1, a growth-factor regulator, reduced fibrosis and improved heart function in a rat model of myocardial infarction (Suresh et al., 2015). Interestingly, MSCs engineered with overexpression of the erythropoietin (EPO) showed significantly enhanced migration *in vitro* in the presence of HGF, as compared with that in the absence of HGF (Wang X et al., 2020). The study by Wang X. et al. (2020) showed that EPO-MSCs improved hepatic fibrosis symptom, including liver function recovery and downregulation of α-SMA and fibronectin in liver. Importantly, EPO-MSCs increased the matrix metalloproteinase (MMP)-9 expression in liver compared with the control, thus suggesting that EPO-MSCs exert their anti-fibrotic potential through a key modulator of ECM degradation by MMPs. Choi J. et al. (2019) conducted the IL-10 gene-edited MSCs to evaluate their therapeutic potential in a mouse liver fibrosis model. The study by Choi J et al. (2019) demonstrated that the genome-edited MSCs overexpressing IL-10 attenuated severe pro-inflammatory responses, inhibited thioacetamide-induced liver fibrosis and ameliorated mouse liver function. The main therapeutic mechanism was mediated by their anti-fibrotic effects of the modified MSCs through suppression of inflammation in the fibrotic liver, such as downregulation of IL-1β, TNF-α and IFN-γ, and inhibition of the activation of hepatic stellate cells (Choi J. et al., 2019), a pericyte-like cell population of the liver as a dominant contributor to liver fibrosis (Mederacke et al., 2013).

Taken together, the natural MSCs and MSC-based therapeutic products have become a promising strategy for reversing fibrosis. Application of engineered MSC-based synergistic approaches can enhance MSC-mediated anti-fibrotic efficacy. MSCs CM, pretreated MSCs, and MSC-derived EVs have emerged as one promising anti-fibrotic treatment option and this is attracting increasing interest. Indeed, the issue of separating these therapeutic approaches as the one successful therapeutic option is challenging and usually a synergistic combination treatment, e.g., GDNF-MSCs-Exos (Chen L et al., 2020), may enhance MSC-mediated anti-fibrotic efficiency. However, the great uncertainty remains for the impact of the fibrogenic environment on the fate and therapeutic properties of MSCs after infusion. The use of MSCs still faces great challenges (as will be discussed later) and, instead, EVs derived from MSCs may be a cell-free alternative for treating fibrotic diseases in preclinical trials. As a prerequisite for clinical application, further preclinical studies for future work toward EVs preparation, dosage, route and duration of administration are necessitated. For better understanding the therapeutic mechanisms, it is important for the analysis of peoteomic profile in EVs to explore which components of EVs are responsible of the therapeutic effects.

### Mesenchymal Stem/Stromal Cells-Based Anti-Fibrotic Clinical Applications

Currently, MSCs are being developed in clinical trials to understand their potential anti-fibrotic properties. We performed a search at the clinicaltrials.gov with key terms (“mesenchymal stem cell,” “mesenchymal stromal cell” and “fibrosis”/“scar”/“keloid”) and 76 MSC-based anti-fibrotic clinical investigations through 27 April 2022 with representative of 5,241 patients totally worldwide were generated (Table 1), of which the 22 trials were completed and one was withdrawn (NCT03058068). Of these, 48 are conducted in liver cirrhosis, 13 in fibrotic scar/keloid, 9 in pulmonary fibrosis, 3 in renal fibrosis, 2 in cystic fibrosis, and one in non-cystic fibrosis bronchiectasis. Natural MSCs derived from different tissues are widely being used in anti-fibrotic clinical applications. One phase I/II trial (NCT04326959) is ongoing to utilize UC-hMSCs/UC-hMSCs CM in patients suffering from keloid. Another phase I/II trial (NCT02786017) has been performing to explore a novel transplantation approach of injectable collagen scaffold combined with UC-hMSCs for patients with decompensated cirrhosis. However, there are no clinical trials registered on the clinicaltrials.gov using the MSCs modified genetically to evaluate their anti-fibrotic clinical applications.

**TABLE 1:** Summary of MSC-based anti-fibrotic clinical trials (*n* = 76, as of 27 April 2022).

Condition	Source	No. of trials	No. of phase I	No. of phase I/II	No. of phase II	No. of phase III	No. of phase IV	No. of phase n/a
Liver cirrhosis	UC-hMSCs	20	4	11	4			1
	BM-hMSCs	11	3	1	5	2		
	AD-hMSCs	3	2					1
	MB-hMSCs	1		1				
	Unknown tissue origin of hMSCs	13		6	2		1	4
Pulmonary fibrosis	UC-hMSCs	2	2					
	WJ-hMSCs	1						1
	BM-hMSCs	2	1	1				
	AD-hMSCs	1		1				
	PD-hMSCs	1	1					
	Unknown tissue origin of hMSCs	2	2					
Renal fibrosis	AD-hMSCs	1						1
	Unknown tissue origin of hMSCs	2		1	1			
Scar/keloid	UC-hMSCs	5	2	1	1			1
	AD-hMSCs	3		2				1
	Unknown tissue origin of hMSCs	5	1	1	2			1
Cystic fibrosis	Unknown tissue origin of hMSCs	2	2					
NCFB	BM-hMSCs	1	1					

AD-hMSCs, human adipose-derived MSCs; BM-hMSCs, human bone marrow-derived MSCs; MB-hMSCs, human menstrual blood-derived MSCs; NCFB, non-cystic fibrosis bronchiectasis; PD-hMSCs, human placenta-derived MSCs; UC-hMSCs, human umbilical cord-derived MSCs; WJ-MSCs, Wharton’s jelly-derived MSCs.

Over the past years, the published clinical studies with MSCs intervention have proposed the therapeutic benefits in patients with the different types of fibrosis. For example, in a previous clinical study, 30 patients with decompensated liver cirrhosis were followed up for 48 weeks after administration of UC-hMSCs (Zhang Z et al., 2012). Clinical data showed that UC-hMSC transfusion can improve liver function and reduce ascite in those patients through evaluation of the model for end-stage liver disease Na scores. Laboratory data from the study by Zhang Z. et al. (2012) presented that a significant downregulation of laminin, hyaluronic acid, and type IV collagen and a significant up-regulation of HGF were observed in treated patients, as compared to the control groups. Clinical phase I/II observations have also proposed that the potential of MSCs as a novel anti-fibrotic cytotherapy approach in other different tissue types of fibrosis including renal interstitial fibrosis (Reinders et al., 2013; Reinders et al., 2015), cardiac segmental fibrogenic scar (Karantalis et al., 2014; Suncion et al., 2014), cesarean section skin scars (Fan et al., 2017; Fan et al., 2018) and vocal fold scar (Hertegård et al., 2020). While application of MSCs is supposed as feasible and safe anti-fibrotic option, the positive, negative, or mixed results have also been observed in anti-fibrotic clinical trials (Yamout et al., 2010; Chambers et al., 2014; Jang et al., 2014). For example, autologous BM-hMSCs administration showed histological improvement in 54.5% patients with alcoholic liver cirrhosis at the week 12 after the second injection (Jang et al., 2014). Likewise, a non-randomized phase Ib trial was to test the safety of dose-escalation human placenta-derived MSCs (PD-hMSCs) infusion at 1 × 10^6^ (*n* = 4) or 2 × 10^6^ (*n* = 4) cells/kg body weight, respectively, in patients with IPF and no significant change was found in the lung fibrosis score over the course of the trial, albeit intravenous MSCs administration was feasible and safe (Chambers et al., 2014). Currently, limited clinical data available to support the strong benefit of MSCs’ application are insufficient and the large trials are needed to examine the safety and effectiveness of MSCs in the anti-fibrotic settings.

## From Pathophysiological Implications to Anti-Fibrosis Properties: The Multifaceted Roles of Mesenchymal Stem/Stromal Cells in Progressive Fibrogenesis

**FIGURE 3:**
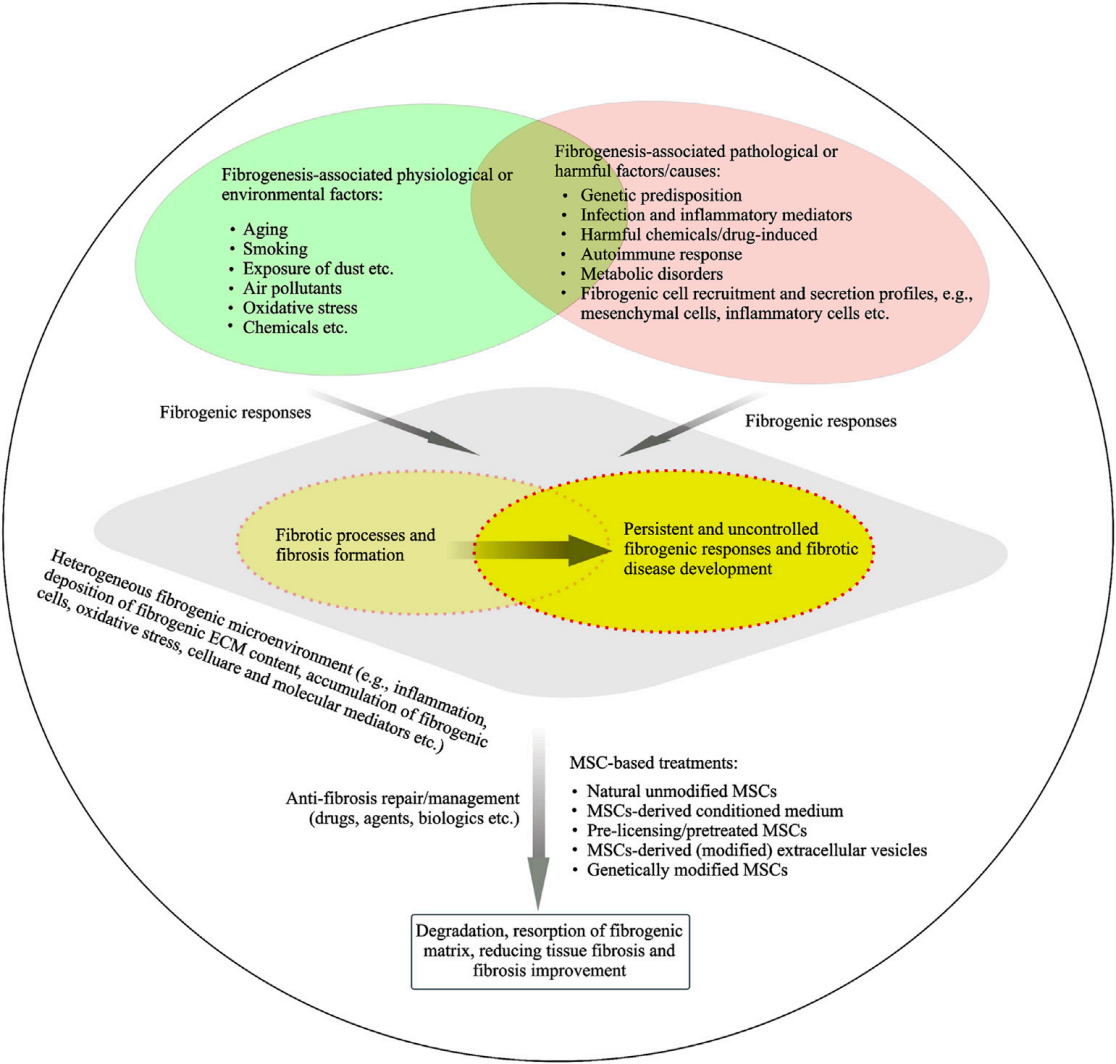
Fibrogenic process and fibrosis resolution under heterogeneous fibrogenic conditions. A hypothetical model of a temporary heterogeneous (pathophysiological) microenvironment is suggested for the understanding of fibrosis physiological or pathological responses. Potential physiological or pathological factors are linked to fibrosis and fibrotic development. MSCs or MSCs derived products are being developed in preclinical and clinical studies to understand their potential anti-fibrotic properties.

There are different types of fibrogenic diseases or fibroproliferative disorders, such as pulmonary fibrosis, liver fibrosis, heart fibrosis, skin fibrosis, kidney fibrosis, cirrhosis and sclerosis, which present different clinical manifestations with a variety of causes. A wide variety of factors/triggers have been recognized for causing fibrogenic formation and development of different fibrotic diseases (Figure 3). Pathological or physiological responses are on the basis of the various contexts in the heterogeneous fibrogenic microenvironments or niches. In this discussion section, we will summarize the multifaceted roles of MSCs in progressive fibrogenesis and address several challenges for MSCs’ translational anti-fibrotic applications.

MSCs play a dual role in pathophysiological and anti-fibrotic implications under certain conditions. For example, as mentioned above, resident MSCs from fibrotic lung tissue have fibrogenic activity and the characteristics of MSCs is dependent on fibrogenic environmental cues. Bonifazi et al. (2020) conducted a previous study to understand the effects of pathological microenvironment on MSCs and the consequent role of MSCs in their pathological implications. This study by Bonifazi et al. (2020) showed that pathological niches modified the biological features of MSCs from IPF lung tissue compared to control MSCs, including the increased expression of molecules related to ECM secretion, inflammation, and oxidative stress. Importantly, IPF lung derived MSCs were able to alter the expression of genes related to inflammation and oxidative stress on the NHLF, a fibroblast lung cell line, after co-culture. Indeed, specific lung-resident MSCs also play a potential role in the regulation of tissue homeostasis (Sveiven and Nordgren, 2020; Samarelli et al., 2021) through their unique immunomodulatory and secretary capacity to provide appropriate tissue-specific niches. MSCs can in part contribute to the recruitment of fibrotic mesenchymal cells (fibroblasts, myofibroblasts and SMCs) through differentiation of MSCs. These mesenchymal cells including MSCs secrete the excessive ECM content in context of pathophysiological fibrosis microenvironment (Figure 3). MSCs act as a potential initiator of coagulation through the release of TF and MSCs retain procoagulant activity to be associated with fibrogenic disease development. TFPI may contribute to the regulation of TF-associated coagulation cascade. MSCs are involved in a fibrinolytic cascade through secreting plasminogen activators under certain contexts. A variety of approaches using MSCs and MSC-based products for targeting anti-fibrotic therapies are described in the current paper.

Given the unique biological properties, MSCs are being explored in the anti-fibrotic treatments and, however, the critical questions and challenges associated with the application of MSCs need to be addressed in the clinical settings. Firstly, there are still debates about the susceptibility of MSCs to spontaneous transformation after long-term *in vitro* culture (Pan et al., 2014; Rodriguez et al., 2014). It is advisable to use the low passage culture of MSCs for safe therapeutic strategies. Secondly, MSCs derived from ectoderm and mesoderm during embryonic development can differentiate towards mesoderm-derived lineages such as osteocytes, adipocytes, chondrocytes (Kumar et al., 2017; Li et al., 2021). Therefore, it is important to emphasize that MSCs are required to maintain the homeostasis of mesenchymal phase to ensure their biological and functional properties. In case of the absence of mesenchymal homeostasis caused by different pathological conditions (e.g., MSCs aging), the stemness/multipotency of MSCs would be lost (Oh et al., 2014; McHugh and Gil, 2018). Therefore, a gold standard needs to be considered to assess the consistency and stability of MSCs in preclinical and clinical applications. Thirdly, preclinical and clinical studies have frequently shown the discrepancy in MSCs anti-fibrotic effects. Clinically relevant animal models with long-term outcomes should be optimized. Fourthly, given a small number of clinical trials currently using MSCs as a potential anti-fibrotic treatment, the optimizing MSCs’ therapeutic regimens have not been formally established. Further optimization of MSC-based anti-fibrotic therapies needs also to be considered in the therapeutic efficacy studies such as the dose and dosing interval of MSCs, the route of administration, the number and timing of MSCs’ administration, the MSCs’ inherent properties, population/subjects and the appropriate endpoint. Finally, several other arguments in MSCs’ application remain to be addressed including the fate of MSCs after infusion, the homing of MSCs, the safety of genetic modification of MSCs, etc.

## Conclusion and Therapeutic Perspectives

Due to the plasticity of MSCs in contribution to fibrosis and anti-fibrotic properties, therapeutic mechanisms of MSCs’ action need to be better understood. Focusing on the multifaceted roles of MSCs in fibrosis, the benefits of MSCs translational applications need to be carefully balanced with MSCs’ potential risks in preclinical and clinical settings, which are being designed to maximize their therapeutic activity while minimizing their potential side effects. For example, triggering of IBMIR is identified potentially by the variability of MSCs’ properties, a high cell dose, an individual MSCs donor, and/or a high passage number. Consequently, optimizing MSCs’ therapeutic regiments require to consider a lower dose of MSCs with low passage number as a more suitable treatment candidate. Testing TF expression and selecting TF-deficient MSCs are necessitated before infusion. Currently, clinical studies using MSCs as an anti-fibrotic therapeutic option are underway and, however, therapeutic benefit in the clinical setting using MSCs is based on their safety and effectiveness of their clinical application. The future clinical settings need to be performed in large and multicenter randomized clinical trials with more patients and long-term follow-ups to assess anti-fibrotic treatment efficacy.

## Glossary

AD-hMSCs: human adipose tissue-derived mesenchymal stromal/stem cells
α-SMA: α-smooth muscle actin
BM-hMSCs: human bone marrow-derived MSCs
CK1δ: casein kinase 1δ
CM: conditioned medium
ECM: extracellular matrix
EMT: epithelial to mesenchymal transition
EPO: erythropoietin
EVs: extracellular vesicles
GDNF: glial-derived neurotrophic factor
HGF: hepatocyte growth factor
IBMIR: instant blood-mediated inflammatory reaction
IGF-I: insulin-like growth factor-I
IPF: idiopathic pulmonary fibrosis
ISCT: International Society for Cellular Therapy
MB-hMSCs: human menstrual blood-derived MSCs
MC: bone marrow concentrate
MSCs: mesenchymal stem/stromal cells
NCFB: non-cystic fibrosis bronchiectasis
PD-hMSCs: human placenta-derived MSCs
PTEN: phosphatase and tensin homolog
SMCs: smooth muscle cells
TF: tissue factor
TFPI: TF pathway inhibitor
TGF-β: transforming growth factor-β
UC-hMSCs: human umbilical cord-derived MSCs
UUO: unilateral ureteral obstruction
VEGF: vascular endothelial growth factor
WJ-MSCs: Wharton’s jelly-derived MSCs
YAP: Yes-associated protein
